# Silencer-delimited transgenesis: NRSE/RE1 sequences promote neural-specific transgene expression in a NRSF/REST-dependent manner

**DOI:** 10.1186/1741-7007-10-93

**Published:** 2012-11-30

**Authors:** Xiayang Xie, Jonathan R Mathias, Marie-Ange Smith, Steven L Walker, Yong Teng, Martin Distel, Reinhard W Köster, Howard I Sirotkin, Meera T Saxena, Jeff S Mumm

**Affiliations:** 1 Department of Cellular Biology and Anatomy, Georgia Health Sciences University, Augusta, GA 30912, USA; 2 Vision Discovery Institute, Georgia Health Sciences University, Augusta, GA 30912, USA; 3 Luminomics, Inc., Augusta, GA 30912, USA; 4 Cancer Center, Georgia Health Sciences University, Augusta, GA 30912, USA; 5 Institute of Developmental Genetics, Helmholtz Zentrum München, Neuherberg, D-85764 Germany; 6 Department of Cellular and Molecular Medicine, University of California San Diego, La Jolla, CA 92093, USA; 7 Zoological Institute, Division of Cell Biology & Physiology, Braunschweig University of Technology, Braunschweig, 38106 Germany; 8 Department of Neurobiology and Behavior, Stony Brook University, Stony Brook NY 11794, USA

**Keywords:** zebrafish, transgenesis, enhancer trap, NRSE/RE1, NRSF/REST, Gal4/UAS, neuron

## Abstract

**Background:**

We have investigated a simple strategy for enhancing transgene expression specificity by leveraging genetic silencer elements. The approach serves to restrict transgene expression to a tissue of interest - the nervous system in the example provided here - thereby promoting specific/exclusive targeting of discrete cellular subtypes. Recent innovations are bringing us closer to understanding how the brain is organized, how neural circuits function, and how neurons can be regenerated. Fluorescent proteins enable mapping of the 'connectome', optogenetic tools allow excitable cells to be short-circuited or hyperactivated, and targeted ablation of neuronal subtypes facilitates investigations of circuit function and neuronal regeneration. Optimally, such toolsets need to be expressed solely within the cell types of interest as off-site expression makes establishing causal relationships difficult. To address this, we have exploited a gene 'silencing' system that promotes neuronal specificity by repressing expression in non-neural tissues. This methodology solves non-specific background issues that plague large-scale enhancer trap efforts and may provide a means of leveraging promoters/enhancers that otherwise express too broadly to be of value for *in vivo *manipulations.

**Results:**

We show that a conserved neuron-restrictive silencer element (NRSE) can function to restrict transgene expression to the nervous system. The neuron-restrictive silencing factor/repressor element 1 silencing transcription factor (NRSF/REST) transcriptional repressor binds NRSE/repressor element 1 (RE1) sites and silences gene expression in non-neuronal cells. Inserting NRSE sites into transgenes strongly biased expression to neural tissues. NRSE sequences were effective in restricting expression of bipartite Gal4-based 'driver' transgenes within the context of an enhancer trap and when associated with a defined promoter and enhancer. However, NRSE sequences did not serve to restrict expression of an upstream activating sequence (UAS)-based reporter/effector transgene when associated solely with the UAS element. Morpholino knockdown assays showed that NRSF/REST expression is required for NRSE-based transgene silencing.

**Conclusions:**

Our findings demonstrate that the addition of NRSE sequences to transgenes can provide useful new tools for functional studies of the nervous system. However, the general approach may be more broadly applicable; tissue-specific silencer elements are operable in tissues other than the nervous system, suggesting this approach can be similarly applied to other paradigms. Thus, creating synthetic associations between endogenous regulatory elements and tissue-specific silencers may facilitate targeting of cellular subtypes for which defined promoters/enhancers are lacking.

## Background

Accurate characterization of neurons and neural circuits requires that neuronal subtypes be unambiguously identified [1] and independently manipulated [2]. Typically, neurons are classified based on morphological (e.g., neurite targeting patterns), molecular (e.g., transmitter expression) and/or physiological properties. Cellular subtypes are defined by both activation and repression of specific gene sets. Repression plays a major role in regulating the expression of neural-specific genes. For instance, the transcriptional repressor neuron-restrictive silencing factor (NRSF)/repressor element 1 silencing transcription factor (REST) serves to silence neural gene expression in non-neural tissues [3,4]. Transgenic techniques provide a means of targeting discrete neuronal subpopulations, affording investigations of the function of specific neuronal cell types and neural subcircuits [5,6]. However, transgenic labeling of neuronal subpopulations in vertebrates has proven to be complicated, as cis-regulatory elements (e.g., promoters, enhancers) that delineate specific neuronal subtypes are sometimes difficult to define. Equally problematic is that many regulatory elements characterized as being cell-type specific within the nervous system actually label cells in other tissues. Optimally, exclusive transgene expression in neuronal cell subtypes is necessary to assign direct causal effects during assays involving gain- or loss-of-function manipulations.

Gene and/or enhancer trap screens, whereby endogenous cis-regulatory elements are co-opted to regulate transgene expression, eliminate the need to identify cell-specific promoters by allowing visual selection of expression patterns of interest. Transposons, such as Tol2, have been used extensively for gene/enhancer trapping in zebrafish (*Danio rerio*) and many of the resultant transgenic lines show expression in the nervous system. However, despite the general success of this approach, so-called basal or background expression in heart, skeletal muscle, etc., can compromise the usefulness of these resources [7]. This has been particularly problematic for enhancer traps employing the Gal4/upstream activating sequence (UAS) bipartite expression amplification system [8-10]. The Gal4/UAS system is based on the yeast transcription factor Gal4, which binds to 17 to 23 bp UAS [11] to drive expression of effector genes/transgenes. A bipartite transgenic system, where Gal4 'drivers' and UAS 'effectors' are derived separately was adapted to the *Drosophila *system [12] as a tissue/cell-specific manipulation platform. Rapid expansion of a set of Gal4 driver lines (specifying where and when transgenes are expressed) and UAS effector/reporter transgenic lines (specifying how much and what transgenes are expressed) soon followed. Driver and effector/reporter lines can be brought together in any combination; the bipartite nature of the Gal4/UAS system thus provides a versatile platform for studies of cell and molecular function that has been employed to great effect within the *Drosophila *community [13]. The source of background expression in zebrafish Gal4/UAS-based transgenic lines has not been resolved. It could be a byproduct of cryptic enhancer elements [8], promiscuous expression as a result of position effects, or otherwise undetectable gene expression that is revealed by the enhanced transcriptional activity of the Gal4/UAS system. The latter possibility is in keeping with interpretations put forth by Fujimoto *et al. *[14], concerning unexpected expression patterns seen in Gal4-VP16 lines (three of three *Tg(optb.A:Gal4-VP16) *lines show previously uncharacterized expression within eye muscles and retinal cells). Regardless of the underlying mechanism, Gal4/UAS lines in which background expression is reduced or eliminated would provide improved resources for functional studies of the nervous system.

Intersectional and subtractive methods, whereby transgenes are restricted to cells expressing two or more patterning genes, have been developed to promote cell-specific expression [15]. Alternatively, transcriptional repression or silencing could be used to delimit transgene expression to specific cell or tissue types. Interestingly, transcriptional repression plays a prominent role in establishing neuronal specific expression patterns [16]. Accordingly, we were interested in determining whether inserting neuronal silencer binding sites into Gal4 driver and/or UAS effector transgenes would serve to restrict transgene expression to the nervous system *in vivo*. This approach could potentially solve Tol2-associated background expression issues with regard to studies of the nervous system in zebrafish. Moreover, synthetic associations between endogenous regulatory elements and tissue-specific silencers may facilitate useful expression patterns not otherwise attainable by standard promoter/enhancer characterizations. Here, we have explored the use of the neuron-restrictive silencer element (NRSE) to delimit transgene expression exclusively to neuronal cells.

Evaluation of regulatory mechanisms underlying neural specificity of the synaptic protein stathmin-like 2 gene (STMN2, also known as SCG10) and a voltage-dependent sodium channel revealed that active repression of expression in non-neuronal cells played a central role [17-19]. An NRSE [20] - also known as restriction element 1 (RE1 [21]) - was identified as being necessary and sufficient for repression of STMN2 and the type II sodium channel. Subsequently, the Krüppel zinc finger protein NRSF/REST was found to bind NRSE/RE1 sites and repress non-neuronal expression of multiple neural-specific genes [3,4]. Upon binding to NRSE/RE1 sites, NRSF/REST acts in concert with a corepressor complex of chromatin remodeling proteins including REST corepressor 1 [22], Sin3A [23] and histone/lysine deacetylases [23,24]. NRSF/REST has been reported to repress target gene expression in both embryonic and neural stem cells [25], whereas corepressors appear to play a role in plasticity of expression in mature neurons [26]. Although NRSE-containing genes can be expressed outside the nervous system - e.g., pancreatic islet cells [27] - and NRSE sites are thought to act as neuronal enhancers in certain contexts [28], the predominant effect of NRSE-mediated gene regulation is to repress the expression of neural genes in non-neuronal cell types. This suggests that integrating NRSE sites into the regulatory elements of transgene constructs might serve to promote specificity by delimiting expression to the nervous system. This approach has shown promise when tested in the context of defined regulatory elements in cell culture [29], viral vectors [30,31] and transgenic assays in mammalian systems [32]. We were interested in whether NRSE sites would be effective for delimiting transgene expression to the nervous system within the context of enhancer trap screens in zebrafish, and thereby provide improved resources for functional studies of the nervous system.

To test this idea, we integrated a pair of consensus NRSE sites [33] into several bipartite transgenic expression system constructs and created corresponding transgenic zebrafish lines. The NRSE site used, TTCAGCACCACGGACAGCGCC, is a canonical NRSE site that is highly conserved across species, and is composed of two non-palindromic half-sites separated by a non-conserved 2 bp spacer (underlined). We placed a tandem set of NRSE sites in upstream regulatory regions of several transgenic constructs and compared resulting expression patterns to non-NRSE parental plasmids. In all, this strategy was applied within the context of enhancer trap constructs [34], defined enhancer constructs [35], defined promoter constructs [36] and UAS-based reporter constructs [37].

The data indicate that transgene expression was strongly biased to the nervous system when NRSE sequences were included in enhancer trap and defined enhancer constructs, thereby effective in delimiting the expression of the driver element of a bipartite expression system (e.g., Gal4-VP16). However, expression biases were not evident when NRSE sequences were added to UAS-based reporter transgenes. Nevertheless, due to the bipartite nature of such systems, delimiting the expression of Gal4-VP16 drivers sufficed to restrict UAS reporters to the nervous system as well - because drivers are required to activate expression of reporters. Morpholino knockdown (this study) and zinc finger nuclease mediated gene disruptions [38] verified that NRSF/REST is required for NRSE-based transgene silencing. Thus, NRSE-delimited transgenesis may help to overcome difficulties in defining cell-specific expression in the nervous system. Accordingly, we are conducting a large-scale enhancer trap screen coupling NRSE sites with bipartite expression systems to facilitate functional manipulations of trapped neuronal cell subtypes. Several other tissue/cell-specific silencer elements have been reported. Examples include a cartilage-specific element [39], a cardiac muscle-specific element [40] and a cell-specific pancreatic silencer element [41]. Thus, transgenes that create novel associations between tissue-specific silencers and more diffusely expressed activator elements may be a useful strategy for labeling and manipulating discrete cell types that are otherwise difficult to demarcate. The existence of tissue-specific silencers in plants [42] suggests such elements exist throughout most multicellular life forms. In summary, silencer-delimited transgenesis may be a broadly applicable strategy that enhances the capacity to exclusively target specific cell types.

## Results

### NRSE-delimited enhancer traps

To test whether associating NRSE sites with a minimal promoter would serve to restrict transgene expression to the nervous system, Gal4-VP16-based enhancer trap constructs were assembled with and without NRSE sites. Plasmids were composed of a mouse *cfos *minimal promoter [34] upstream of an optimized Gal4-VP16 transcriptional activator, termed KalTA4 [43], and assembled within the miniTol2 cassette [44]. Initially, transient transgenesis assays were used to compare control plasmids (CK, *cfos:KalTA4*; see construct diagrams in Additional file 1) and test plasmids having a tandem repeat of two NRSE sites inserted 18 bp upstream of the minimal promoter (NRCK, *2xNRSE-cfos:KalTA4*). CK or NRCK transgenes were injected into fertilized eggs from an established UAS effector-reporter line, (14xNTR-Ch, *Tg(14xUAS-E1b:nfsB-mCherry)c264 *[45]) and resulting mCherry expression patterns monitored daily until 6 to 7 days post-fertilization (dpf). These preliminary results suggested that the NRSE-containing plasmid, NRCK, was preferentially expressed in neural tissues (data not shown). Following these studies, we created multicistronic 'self-reporting' enhancer trap constructs linking the Gal4-VP16 driver and UAS reporter directly within a single transgene (i.e., in *cis*). In the NRSE version, a *loxP *flanked 5xUAS:YFP reporter was placed downstream of NRCK (NRCK-5xMY, *2xNRSE-cfos:KalTA4, loxP-5xUAS-E1b:gap43-EFYP-loxP*). The control version consisted of the CK driver element upstream of tandem *5xUAS:nfsB *effector and *14xUAS:YFP *reporter components (CK-5xN-14xY, c*fos:KalTA4, 5xUAS:Eco.nfsB, 14xUAS:gap43-YFP*), as previously reported [46].

**Figure 1:**
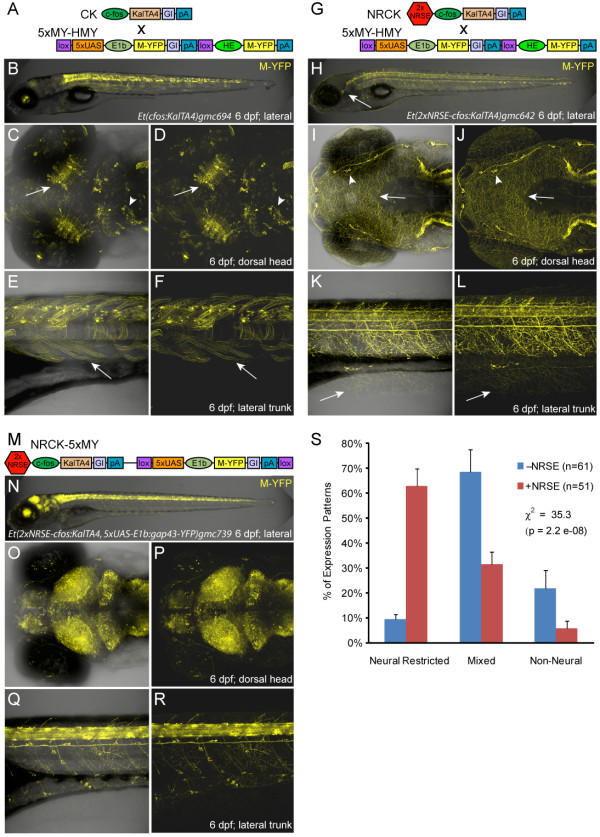
**Enhancer trap comparisons ±NRSE**. Plasmid diagrams and representative confocal images of Gal4 expressing enhancer trap lines ±NRSE, one CK (A-F) and two types of NRCK (G-L and M-R) lines are shown (6 dpf). **(A) **CK driver and 5xMY-HMY reporter plasmid diagrams. **(B-F) **The majority of CK lines showed expression in multiple tissues (B), with neural expression in regions such as the tectum (C, D, arrows) and midbrain (C, D, arrowheads) as well as labeling of skeletal muscle (E, F, arrows). **(G) **NRCK driver and 5xMY-HMY reporter plasmid diagrams. **(H-L) **The majority of NRCK lines displayed neuronally restricted expression patterns (H), dense innervation patterns in the skin indicate somatosensory neuron labeling (H-L, arrows), neuromast innervation is also evident in this line (I, J, arrowheads). **(M) **NRCK-5xMY plasmid diagram. **(N-R) **Like NRCK lines, the majority of NRCK-5xMY 'self-reporting' lines displayed neuronally restricted expression. The density of reporter labeling was typically high with near ubiquitous expression throughout the central nervous system being common (O-R). **(S) **Quantification of phenotypes from non-NRSE (-NRSE, blue bars, total of 61 lines) and NRSE-containing enhancer trap lines (+NRSE, red bars, total of 51 lines). Expression patterns were classified as either neural-restricted (as in H-L and N-R), mixed (as in B-F), or non-neural (example not shown). The data shows a clear trend toward neuronally restricted expression patterns when NRSE elements are included in enhancer trap transgenes. To evaluate the significance of this trend, chi-square tests of independence were performed comparing the ±NRSE phenotyping datasets. The results show clear statistical differences across all lines ±NRSE (values shown in graph) and between matched subsets (CK versus NRCK, χ^2 ^= 18.5, *P *= 9.8e^-05^; CK-5xN-14xY versus NRCK-5xMY, χ^2 ^= 16.4, *P *= 2.7e^-04^). The error bars indicate the standard deviation in phenotype between the two +NRSE and -NRSE transgenic lines.

To more stringently test the effects of NRSE sites on transgene expression patterns, CK, NRCK and NRCK-5xMY plasmids were used to establish a series of stable enhancer trap lines (Figure 1). Individual plasmids were injected along with Tol2 transposase mRNA into UAS:reporter-containing eggs derived either from 14xNTR-Ch or 5xMY-HMY lines (see below). Reporter-expressing embryos/larvae were raised to sexual maturity then mated to test for germline transmission. Reporter-expressing progeny were raised as filial generation one (F1) lines. Subsequent propagations were used to separate out individual lines according to unique and reproducible expression patterns - i.e., to account for multiple transgene integrations. The total number of enhancer trap lines generated to date is: 24 CK (*Et(cfos:KalTA4)*), 25 NRCK (*Et(2xNRSE-cfos:KalTA4)*) and 26 NRCK-5xMY (*Et(2xNRSE-cfos:KalTA4, 5xUAS-E1b:gap43-YFP)*), as well as 37 CK-5xN-14xY (*Et(2xNRSE-cfos:KalTA4, 5xUAS-E1b:nfsB, 14xUAS-E1b:tagYFP*)). These lines have been propagated to the F3 to F5 generation and 93% currently produce inheritance patterns consistent with a single insertion site (at least with regard to visually detectable integration events). In addition to the data presented here, we have established a website for the dissemination of high-resolution imaging data and insertion site sequence information to the research community [47]. We continue to generate new data regarding these and related transgenic lines and thus plan to expand the data available on this site on an ongoing basis.

Initial evaluations of transgene expression patterns were promising. However, we noted that mCherry patterns often seemed unstable, that is, expression domains would differ slightly from one generation to the next. It was subsequently reported that the *Tg(14xUAS-E1b:nfsB-mCherry)c264 *line is susceptible to methylation-based silencing [48,49]. To avoid NRSE-independent silencing issues, which could obviously compromise our expression pattern analyses, we created a new 5xUAS reporter line expressing membrane-tagged yellow fluorescent protein (YFP; 5xMY-HMY, *Tg(loxP-5xUAS-E1b:gap43-YFP-loxP, he1a:gap43-YFP)gmc930*, see Additional file 1). Importantly, the 5xMY-HMY (*gmc930*) line has shown no evidence of silencing over three generations, possibly due to our inclusion of 'barrier' insulator sequences [50] or the reduction in the number of repetitive UAS elements [49]. Barrier insulators are thought to function by maintaining histones of chromatin surrounding the site of transgene integrations in a hyperacetylated state, thereby blocking encroachment of heterochromatin [51]. Either way, the 5xMY-HMY line provided an improved resource for defining expression patterns of CK and NRCK enhancer trap lines. All subsequent expression analyses were performed by crossing KalTA4 enhancer trap lines to 5xMY-HMY and/or by creating driver lines directly in 5xMY-HMY fertilized eggs.

Phenotypic characterizations of KalTA4 driver lines at 6 to 7 dpf revealed clear differences between control (CK) and NRSE-containing (NRCK) expression patterns. CK-derived lines tended to have mixed expression patterns, with multiple cell types and tissues labeled (Figure 1A-F, see Additional file 2 or the web-based database (referenced above) for more images of CK lines). The CK *gmc694 *line provides a typical example, with expression in tectal neurons (Figure 1C, D, arrows), midbrain neurons (Figure 1C, D, arrowheads) and skeletal muscle (Figure 1E, F, arrows). By contrast, expression in NRCK lines tended to be restricted to neural tissues (Figure 1G-L, see Additional file 2 or the web-based database for more images of NRCK lines). As an example, the NRCK *gmc642 *line displays strong expression in somatosensory neurons. In particular, expression is seen in the trigeminal ganglion posterior to the eye (Figure 1H, arrow), cranial sensory neurons (Figure 1I, J), and Rohon-Beard cells of the spinal cord (Figure 1K, L). Dense innervation throughout the skin of the head (arrow in Figure 1I, J, arrowhead denotes innervation of sensory placode) and trunk regions (Figure 1K, L, from Rohon-Beard cells, arrow) is easily visualized due to the membrane-tagged YFP reporter. This same trend held for self-reporting NRCK-5xMY lines (Figure 1M-R). However, the level of reporter expression tended to be markedly stronger in NRCK-5xMY self-reporting lines than when NRCK was crossed to 5xUAS:YFP lines. For instance, the NRCK-5xMY *gmc739 *line has near ubiquitous expression throughout the central nervous system, as seen in the brain (Figure 1N-P) and spinal cord (Figure 1Q-R). Similar differences in the level of expression between Gal4-VP16 and UAS transgenes integrated in *cis *versus in *trans *have been previously observed [43]. The mechanism underlying this phenomenon is not entirely clear but could be due to effects that trapped enhancers have on both the *cfos *and *E1b *(i.e., UAS-associated) minimal promoters. By contrast, non-NRSE self-reporting CK-5xN-14xY controls [46] produced predominantly mixed expression phenotypes (see Table 1 for quantification).

**Table 1:** Phenotype characterization of NRSE versus non-NRSE enhancer trap lines.

Lines	Percentage neural-restricted (number/total)	Percentage mixed (number/total)	Percentage non-neural (number/total)
CK	8% (2/24)	75% (18/24)	17% (4/24)
CK-5xN-14xY	11% (4/37)	62% (23/37)	27% (10/37)
NRCK	68% (17/25)	28% (7/25)	4% (1/25)
NRCK-5xMY	58% (15/26)	35% (9/26)	8% (2/26)
C1CK-5xY2N	13% (1/8)	75% (6/8)	13% (1/8)
NRC1CK-5xY2N	82% (9/11)	18% (2/11)	0% (0/11)

**Totals**			

**- NRSE Tg**	**10% (7/69)**	**68% (47/69)**	**22% (15/69)**
**+ NRSE Tg**	**66% (41/62)**	**29% (18/62)**	**5% (3/62)**

To quantify the effect of inserting NRSE sites into enhancer trap transgenes, KalTA4 driver lines were classified between 6 and 9 dpf as displaying one of three phenotypes: neural-restricted, mixed and non-neural. The results show NRSE-containing driver lines (including self-reporting NRCK-5xMY lines) exhibit a clear bias toward neural-restricted expression compared with controls (Figure 1S, Table 1). Of 62 NRSE driver lines evaluated, 66% (41 lines) were classified as having neural-restricted expression. Conversely, of 69 characterized control lines, only 10% (seven lines) showed a neural-restricted pattern. Lines showing mixed expression patterns (see Figure 1B-F) made up 68% of CK controls (47 lines) and 29% of NRSE drivers (18 lines). Non-neural lines made up 22% of controls and only 5% of NRSE lines (Table 1). These data suggest that NRSE sites can have dramatic effects on transgene expression patterns within the context of endogenous enhancer elements trapped by a *cfos *minimal promoter. Due to the bipartite nature of the Gal4/UAS system, NRSE-delimited expression of an optimized Gal4-VP16 transcriptional activator translated to neural-restricted expression of a UAS:YFP reporter whether Gal4-VP16 and UAS transgenes were in *trans *(Figure 1G-L) or *cis *(Figure 1M-R) orientations.

### NRSE-delimited expression of a defined enhancer

**Figure 2:**
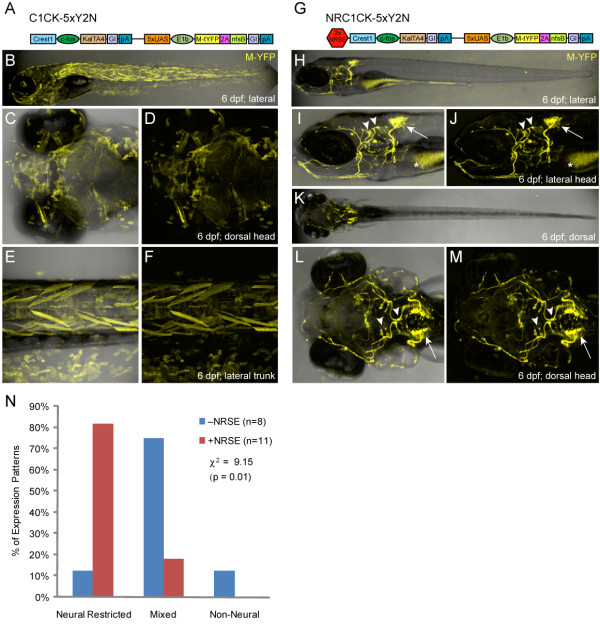
***CREST1-cfos *transgene comparisons ±NRSE**. Plasmid diagrams and representative confocal images of lines derived with the CREST1-cfos enhancer-minimal promoter element ±NRSE, one C1CK-5xY2N (A-F) and one NRC1CK-5xY2N (G-M) line is shown (6 dpf). **(A) **C1CK-5xY2N plasmid diagram. **(B-F) **The majority of C1CK-5xY2N lines showed mixed expression patterns (B), with labeling of epidermis, skeletal muscle and notochord often evident (C-F). **(G) **NRC1CK-5xY2N plasmid diagram. **(H-M) **The majority of NRC1CK-5xY2N lines had expression restricted to a cranial motor neuron subpopulation (H) - the expression pattern originally characterized as CREST1-specified [35] - including cranial nerve X neurons in the hindbrain (arrows, I, J and L, M) and cranial nerve VII and V neurons in the midbrain (arrowheads, I, J and L, M). Autofluorescence in the gut is marked by asterisks (I, J). **(N) **Quantification of phenotypes from non-NRSE (-NRSE, blue bars, total of eight lines) and NRSE-containing (+NRSE, red bars, total of 11 lines) CREST1-cfos lines; expression patterns were classified as either neural-restricted (as in H-M), mixed (as in B-F), or non-neural (example not shown). The data shows a clear trend toward neuronally restricted expression patterns when NRSE elements are included in CREST1-cfos transgenes. A chi-square test of independence showed significant differences between the ±NRSE phenotyping datasets (values shown in graph).

To extend our evaluation of NRSE-containing transgenes, we next turned to a defined enhancer element from the *Islet1 *locus, termed CREST1, which had proved problematic for our studies. An 800 bp CREST1 sequence was previously characterized as an enhancer element sufficient to direct transgene expression to cranial motor neurons [35]. We were interested in using CREST1 to generate transgenic models to study cranial motor neuron regeneration using the nitroreductase-based system of targeted cell ablation [52-54]. Unfortunately, when CREST1 was inserted into a self-reporting KalTA4/5xUAS-based construct expressing both tagYFP (Evrogen; Moscow, Russia) and nitroreductase (i.e., *CREST1-cfos:KalTA4, 5xUAS-E1b:tagYFP-2A-nfsB*, C1CK-5xY2N, Figure 2A and Additional file 1), we found that enhanced expression from the Gal4/UAS elements resulted in a loss of expression specificity in stable lines (Figure 2B-F). However, when NRSE sites were inserted upstream of the CREST1 enhancer (*2xNRSE-CREST1-cfos:KalTA4, 5xUAS-E1b:YFP-2A-nfsB*, NRC1CK-5xY2N, Figure 2G and Additional file 1), cranial motor neuron-specific expression was restored (Figure 2H-M). The distinctive projection patterns of branchiomotor neurons of the V^th^, VII^th ^and X^th ^cranial nerves [55] made it easy to verify NRSE-delimited expression was specific to the targeted subpopulations (Figure 2I, J and Additional file 3). We quantified these results by characterizing all stable transgenic lines derived with these two constructs as per the phenotypic criterion established above. The data showed that inclusion of the NRSE sequences dramatically improved expression specificity of the previously characterized motor neuron enhancer (Figure 2M, Table 1). Of the 11 NRSE-containing CREST1 lines, 82% (nine lines) showed neural-restricted expression (i.e., neuron-specific expression), while 18% (two lines) had mixed expression; no line displayed a non-neural phenotype. Conversely, non-NRSE lines predominantly displayed mixed expression (75%, six of eight lines), with one line showing neural-restricted and another showing non-neural expression. These data support previous findings and show that neural-specific expression from a defined enhancer can be reinforced by including NRSE sites, particularly if the use of expression amplification systems such as Gal4/UAS results in a loss of specificity.

### NRSE sites fail to restrict UAS transgene expression patterns

**Figure 3:**
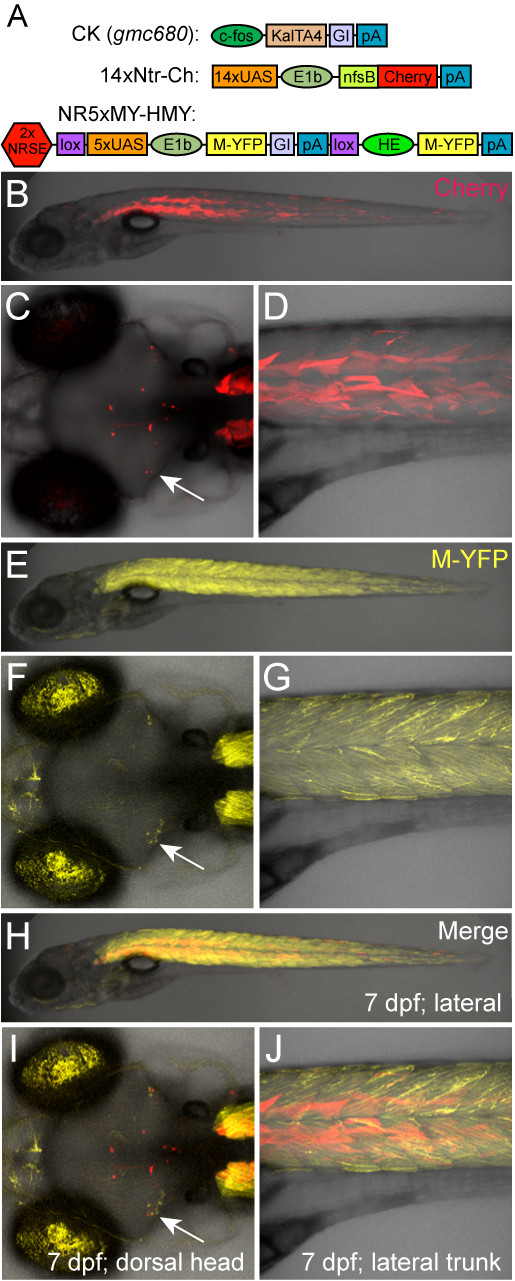
**UAS reporter comparison ±NRSE**. Plasmid diagrams and representative confocal images at 6 dpf of a triple transgenic larva where Gal4 is driving two reporters ±NRSE, a non-NRSE mCherry reporter line (B-D, 14xNTR-Ch (*c264*)) and a NRSE-containing YFP reporter line (E-G, NR5xMY-HMY (*gmc932*), merged images H-J). **(A) **CK, 14xNtr-Ch and NR5xMY-HMY plasmid diagrams. **(B-D) **The control mCherry reporter (non-NRSE) shows a typical mixed expression pattern with neuronal subsets (C, arrow) and skeletal muscle labeling (D). **(E-G) **The NRSE-UAS YFP reporter showed expanded expression in skeletal muscle (compare D and G) and a similar pattern in the brain (F, arrow). Note that autofluorescence from iridiphores in the eye is also evident in the YFP emission channel. **(H-J) **Merged image showing that the addition of NRSE sites to UAS reporter plasmids is not sufficient to restrict reporter expression to the nervous system; i.e., the subset of neurons labeled in the brain (I, arrow). Presumably due to reduced silencing of the NR5xMY-HMY line, there is actually an expansion of YFP reporter expression throughout skeletal muscle.

Encouraged by results obtained with KalTA4 driver lines, we were interested to determine whether NRSE sites could limit expression of UAS reporter transgenes to the nervous system. If NRSE sites could be shown to work within the context of UAS transgenes as well (i.e., when incorporated near UAS elements), this would serve to restrict effectors/reporters to the nervous system regardless of the Gal4-VP16-specified pattern. In turn, this would provide a means of eliminating the background issues of previously derived Gal4-VP16 expressing transgenic lines - a possibility worth exploring given the numerous intriguing neural expression patterns characterized in previous gene/enhancer traps [8-10]. Accordingly, we constructed two versions of our 5xUAS:M-YFP core reporter transgene, one without and one with NRSE sites. The non-NRSE version was the control construct used to establish the 5xMY-HMY line used above (see Figure 1 and Additional file 1). The second transgene contained the tandem NRSE repeat just upstream of the 5xUAS sequence (*2xNRSE-loxP-5xUAS-E1b:gap43-YFP-loxP, he1a:gap43-YFP*, NR5xMY-HMY). Both were constructed in the miniTol2 vector [44] and used to establish stable lines identically to the previously described technique. To test whether the addition of NRSE sites served to neuronally restrict reporter expression, a double transgenic CK enhancer trap line (*Et(cfos:KalTA4)gmc680; Tg(14xUAS-E1b:nfsB-mCherry)c264*) was crossed to an NR5xMY-HMY line (*Tg(loxP-2xNRSE-5xUAS-E1b:gap43-YFP-loxP, he1a:gap43-YFP)gmc932*) to create triple transgenic offspring (Figure 3A). The CK-based *gmc680 *enhancer trap line was derived in the 14xNTR-Ch reporter background and initially characterized as having broad expression in muscle and in a few sparse neurons within the tectum and midbrain regions (Figure 3B-D). We reasoned that if the NRSE-UAS linkage caused reporters to be restricted to neurons, then crossing the CK *gmc680 *enhancer trap line to the NR5xMY-HMY *gmc932 *line should result in the YFP reporter being expressed in the subset of neurons labeled but repressed in muscle. However, we found that this was not the case (Figure 3E-J). Instead, YFP was expressed throughout an even wider swath of muscle cells, as well as labeled neurons (Figure 3F and 3I, arrow). Similar results were obtained when other enhancer trap lines were crossed with NR5xMY-HMY reporter lines (data not shown). Together, these findings suggest that NRSF/REST binding is not able to disrupt transcriptional activation resulting from Gal4-VP16 interactions with UAS elements. Thus, to be effective for Gal4/UAS-based gene/enhancer trapping, NRSE sites need to restrict Gal4-VP16 expression (i.e., be associated with the minimal promoter of the driver, not UAS elements of the effector/reporter).

### Temporal aspects of NRSE-delimited transgenesis

**Figure 4:**
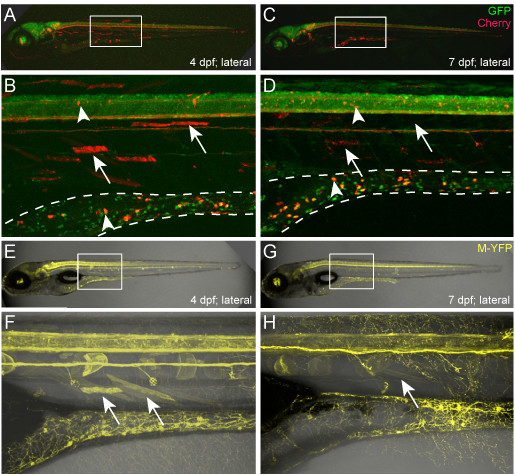
**Delayed repression of non-neural transgene expression**. (**A-H) **Representative time series confocal images of 4- and 7-dpf transgenic larvae showing gradual loss of reporter expression from non-neural tissues; a common phenotype of NRSE-delimited Gal4-VP16 driver lines. **(A-D) **Time series of a triple transgenic larva from a cross between an NRSE-delimited Gal4-VP16 (KalTA4) driver line (NRCK *gmc607*), an mCherry reporter line (*Tg(14xUAS:nfsb-mCherry)c264*), and a pan-neuronal marker for neuronal expression, *Tg(elavl3:EGFP)knu3 *line (formerly *HuC:GFP *[56]). Image magnifications of bordered regions in A and C show skeletal muscle expression fading over time (B and D, arrows), while neuronal expression is maintained (B and D, arrowheads). Expression within the gut (dashed line) is an enteric neuron subpopulation; note 100% overlap with the pan-neural *elavl3:GFP *reporter (co-expressing cells appears yellow). **(E-H) **Time series of a double transgenic larva from a cross between an NRSE-delimited Gal4-VP16 (KalTA4) driver line (NRCK *gmc607*) and the YFP-expressing reporter line (5xMY-HMY *gmc930*). Image magnifications of bordered regions in E and G show skeletal muscle expression also fades over time when driver lines are crossed to YFP reporter lines (F and H, arrows), thus this phenomenon is not a result of NRSE-independent silencing of the reporter (as has been shown for the *Tg(14xUAS:nfsb-mCherry)c264 *line [48,49]). YFP expression typically takes longer to decay than mCherry, possibly due to stronger basal expression and/or absence of methylation due to the reduced number of UAS elements [49] or barrier insulator sequences. Note that the membrane-tagged YFP reporter improves visualization of axonal and dendritic neuronal outgrowths, e.g., the labeled enteric neuron subpopulation in the gut.

During the course of these studies, we were interested in identifying enhancer trap lines that labeled distinct neuronal subtypes. Accordingly, we screened F1 larvae for early and late stage transgene expression (1 to 5 dpf). In addition to revealing lines that express reporters only after terminal neuronal differentiation has begun (3 to 5 dpf), we also observed that some NRSE-delimited lines exhibited expression in muscle cells early on, which then faded over time - i.e., a 'delayed non-neuronal repression' phenotype. The *Et(2xNRSE-cfos:KalTA4)gmc607 *line provides an example of this phenomenon (Figure 4). At 4 dpf, neuronal and skeletal muscle expression is evident in this line (Figure 4A, B, arrows indicate muscle cells). Overlap with the pan-neuronal labeled line, *Tg(elavl3:EGFP)knu3 *(formerly *HuC:GFP *[56]) was used to demarcate which mCherry 'trapped' cells are neuronal (Figure 4A, B, arrowheads). When the same larvae were imaged three days later (7 dpf), muscle cell expression had faded, fragmenting with time and eventually disappearing (Figure 4C, D, arrows indicate fragmented cells), while neuronal expression in the spinal cord and gut was maintained (Figure 4C, D, arrowheads). As noted above, to ensure that this phenotype was not due to NRSE-independent silencing of the UAS:mCherry reporter line, all KalTA4 driver lines were re-evaluated by crossing to the 5xUAS:YFP reporter line (5xMY-HMY*gmc930*). This analysis showed that the delay in non-neuronal repression we observed with the Cherry line was also evident when such lines were crossed with the YFP reporter line (Figure 4E-H). In keeping with its longer half-life, the time required for loss of non-neural expression of YFP was extended in some cases (e.g., to 9 to 11 dpf). In all, we observed the delayed non-neuronal repression phenotype - early non-neural expression of reporter which then fades with time - in 14 of 32 (44%) NRSE enhancer trap lines. Nevertheless, when characterized between 6 and 11 dpf, the majority of enhancer trap lines presented a neural-restricted expression phenotype.

### Changes in REST expression are correlated with delayed transgene repression

**Figure 5:**
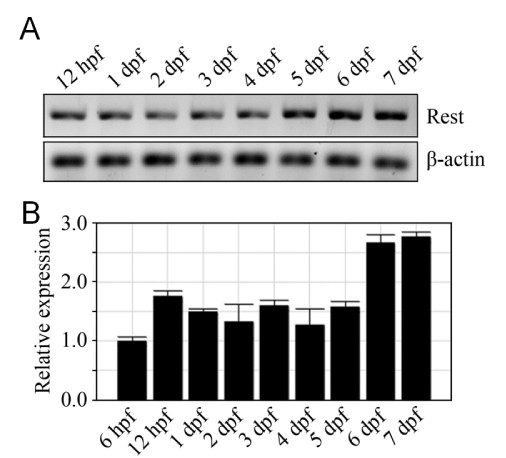
**qRT-PCR analysis of developmental REST expression**. (**A) **Electrophoresis gel of RT-PCR products showing relative levels of REST (top) and β-actin (bottom) expression at 12 hours post-fertilization and then daily from 1 to 7 dpf. **(B) **Graph showing normalized REST expression levels relative to β-actin controls as assayed by qRT-PCR. This analysis shows that REST expression increases significantly at 6 dpf, a time point correlated with reductions in skeletal muscle expression in NRSE-containing enhancer trap lines. Relative expression analyses were performed using 2-ΔΔCT methods; the experiment was repeated a total of three times, with averages and standard deviations shown per time point.

NRSF/REST expression is complex, displaying stage- and neuronal cell-type specific expression patterns and splice variants that reflect a diversity of roles in regulating gene expression. In zebrafish, NRSF/REST expression has only been characterized through early embryonic stages where it is expressed fairly ubiquitously throughout the nervous system until downregulated in differentiating ventrolateral domains of the central nervous system [57]. This pattern is somewhat inconsistent with the early neural expression we observed in the majority of neural-restricted enhancer trap lines (Additional file 4). However, because REST protein levels are modulated post-translation [58,59], protein expression domains may not correlate well with mRNA expression domains. Nevertheless, our data are consistent with the possibility that NRSF/REST may also play a role in activating expression in discrete neural cell subtypes, as suggested in studies of the REST4 isoform in the mouse [60]. Another possible explanation would be that the repressive function of NRSF/REST requires a threshold of expression. This might also explain the delayed repression phenotype seen in some NRCK enhancer trap lines (see Figure 4). To address this question we evaluated NRSF/REST mRNA expression levels by qRT-PCR from 6 h to 7 dpf. Interestingly, expression levels increased approximately two-fold at 6 dpf (Figure 5A, B), coinciding with the time we see evidence of reporter loss in non-neural cells in lines displaying delayed repression. It is also possible the repressive function of NRSF/REST requires co-factors that are not expressed until later stages of development. Finally, subcellular localization changes [61,62] alter NRSF/REST function during development as well. To provide more clarity on this issue, we used morpholino knockdown and zinc finger nuclease targeting [38] to determine whether NRSF/REST expression was required for early neural expression and/or the delayed non-neural repression phenotypes.

### NRSE-mediated transgene repression is dependent on NRSF/REST expression

**Figure 6:**
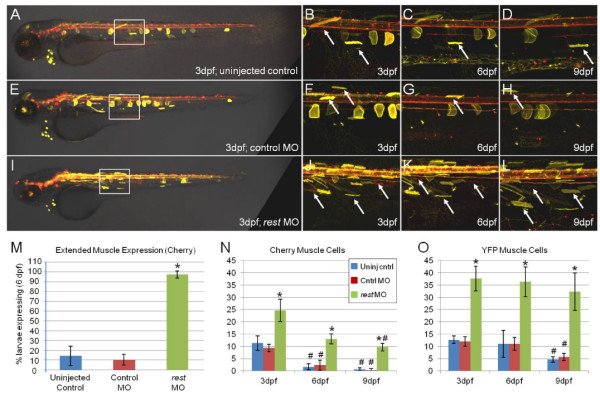
**NRSE-delimited transgene expression is REST dependent**. A-L show representative time series confocal images at 3, 6 and 9 dpf of NRCK; 14xNTR-Ch; 5xMY-HMY triple transgenic larvae ± *rest *morpholino (MO) injection. **(A-D) **Uninjected control larvae showed the delayed repression phenotype of NRCK line *gmc607*, where early skeletal muscle expression (A, B, arrows) fades over time (C, D). Note that loss of the YFP reporter is delayed relative to the mCherry reporter. **(E-H) **Control MO-injected larvae display the same phenotype as uninjected controls with muscle expression (E, F, arrows) fading over time (G, H). **(I-L) **Conversely, *rest *MO-injected larvae have an expansion of skeletal muscle expression at day 3 (I, K, arrows) that fails to be repressed with time (K, L, arrows). **(M-O) **Quantification of phenotypes between uninjected, control MO, and *rest *MO-injected larvae; the number of larvae with detectable mCherry expression in muscle cells at 6 dpf was increased for *rest *morphants (green bar) relative to controls (M, asterisk *P *< 0.01). The number of mCherry (N) and YFP expressing (O) muscle cells also increased for *rest *morphants (green bars) relative to uninjected (blue bars) and MO controls (red bars) at all time points (N, asterisks *P *≤ 0.01, number symbols *P *< 0.05; O, asterisks *P *< 0.001, number symbols *P *< 0.05). Morpholinos were injected at 0.25, 0.5, 1 and 2 μM with similar results (data from representative 0.5-μM injections is shown, experiment was repeated six times). Statistical comparisons were performed using an independent sample *t*-test to compare across treatment conditions per time point (asterisks), and a repeated measures *t*-test to compare individual samples to their initial values at 3 dpf (number signs).

To determine if NRSF/REST expression is required for NRSE-mediated effects on transgene expression, we evaluated the delayed repression phenotype following disruption of NRSF/REST expression. A splice junction targeted morpholino-modified oligonucleotide (MO) against zebrafish *rest *mRNA [57] was injected into NRCK; 14xNTR-Ch; 5xMY-HMY triple transgenic eggs displaying the delayed reporter loss phenotype (*gmc607*). Comparisons among uninjected control, control MO and *rest *MO-injected larvae showed clear differences in reporter expression (Figure 6). Uninjected (Figure 6A-D) and control MO-injected (Figure 6E-H) larvae displayed the expected delayed repression phenotype, with loss of muscle expression over time. By contrast, the number of muscle cells expressing reporter proteins appeared to be increased at 3 dpf in larvae injected with *rest *MO (Figure 6I, J). In addition, muscle cell reporter expression was maintained in *rest *MO-injected larvae at 6 and 9 dpf (Figure 6K, L), well after the sparse muscle expression evident in 3-dpf control larvae had dissipated. As an additional control, *rest *MO injections had no effect on transgene expression when enhancer trap lines without NRSE insertions were tested (i.e., CK;5xMY-HMY double transgenic lines). The number of larvae expressing the mCherry reporter in muscle cells at 6 dpf was quantified across all three conditions (Figure 6M). A total of 96% (44 out of 46) of the *rest *MO group continued to express detectable reporter in muscle cells, whereas only 11% (6 out of 54) in the uninjected and 8% (3 out of 37) in control MO groups did so. Furthermore, when the number of muscle cells expressing reporter protein at 3, 6 and 9 dpf was quantified from all imaged larvae, *rest *MO-injected larvae showed clear increases in muscle cell expression compared to controls (Figure 6N, O). These results strongly suggest that REST expression is required to repress non-neural expression of NRSE-containing transgenes. This same trend held for 1-μM and 2-μM MO-injection experiments over a total of seven independent MO assays. Similar results were obtained from analyses of NRCK transgenic lines crossed into a newly characterized *rest *mutant background [38]. Together, these studies show NRSE/RE1 sequences promote neural-specific transgene expression in a NRSF/REST-dependent manner.

## Discussion

**Figure 7:**
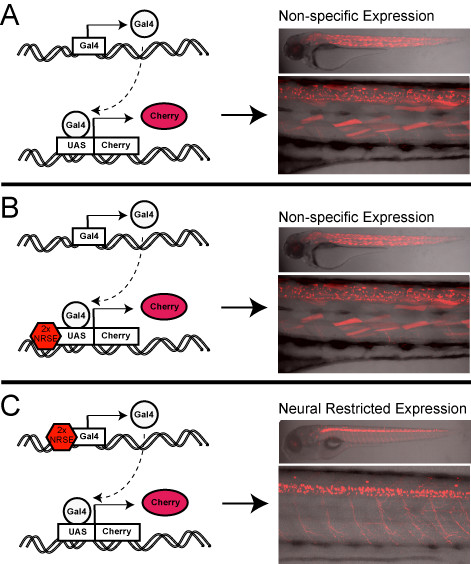
**Summary**. Schematic summarizing our findings regarding NRSE-delimited transgene expression within the context of bipartite driver (B) and effector/reporter lines (C). In each panel, the driver transgene (e.g., Gal4-based) is at the top and the reporter/effector (e.g., UAS) is at the bottom. Transgene products Gal4 and Cherry are shown as circles and ovals, respectively. **(A) **In the absence of NRSE sites, bipartite driver systems are prone to broad non-specific expression patterns with evidence of so-called background expression in skeletal muscle and heart (see Figures 1 and 2). **(B) **Associating NRSE sites with effector/reporter transgenes is not sufficient to restrict transgene expression to the nervous system (see Figure 3), possibly due to an inability to overcome enhanced transcriptional activity typical of artificial bipartite drivers (e.g., Gal4-VP16). **(C) **Incorporating NRSE sites into driver transgenes serves to bias expression toward neuronal specific patterns (see Figures 1 and 2). These findings suggest that creating novel associations between regulatory activators (e.g., enhancers) and silencer elements (e.g., NRSE sites) is a useful strategy for attaining tissue-specific expression patterns that extend beyond what can be obtained with standard transgenesis techniques.

We have explored the use of tissue-specific silencer elements to delimit transgene expression to a region of interest by repressing transcription elsewhere. This strategy provides a potential means of eliminating unintended expression (e.g., cryptic background patterns) and/or undesirable expression (e.g., fine-tuning promoters that target desired cell types as well as other tissues). In particular, we tested whether inserting NRSE sites into upstream regulatory regions of Gal4-VP16 driver and/or UAS-based reporter constructs (Additional file 1) would serve to delimit transgene expression to the nervous system. We found that inclusion of tandem NRSE sites strongly biased the expression of Gal4-VP16 driver transgenes to the nervous system (Figures 1 and 2) but did not restrict expression when associated solely with UAS reporter transgenes (Figure 3). Nevertheless, due to the binary nature of the Gal4/UAS system, restricting Gal4-VP16 driver expression is sufficient to limit UAS-linked effector/reporters to Gal4-VP16 expression domains when the two components are brought together (Figures 1 and 2, summarized in Figure 7). Thus, although NRSE-UAS vectors do not appear adequate to alter existing Gal4-VP16 resources, new NRSE-Gal4-VP16 lines can be derived to provide an improved resource for manipulating neural tissues. By providing improved spatial control over molecules that manipulate neural cell function (e.g., dominant-negatives, optogenetic tools, etc), NRSE-delimited toolsets will enhance the capacity to ascribe cause and effect relationships to cellular and/or molecular function.

Adaptation of the Gal4/UAS system to the zebrafish system [37] was initially met with great enthusiasm due to the versatile and powerful nature of bipartite transgene expression systems. However, this effort soon became fraught with problematic issues, such as low expressivity, Gal4-VP16 toxicity and, more recently, methylation-based transgene silencing of UAS reporter lines. Solutions to these problems have been developed [43,49,63,64], as well as strategies for Gal4-VP16-based lineage tracing [43]. However, a general lack of cell-specific expression of many of the Gal4-VP16 driver lines generated to date presents an obstacle to the widespread deployment of such resources. Efforts to address this issue have included the development of a miniTol2 cassette [44], characterizations of alternative minimal promoters [10,43,64,65], the use of gene traps as opposed enhancer traps [8], and modulating Gal4 expression through interactions with Gal80 [14,66]. We reasoned that an alternative strategy would be to leverage tissue-specific silencer elements to delimit transgene expression to tissue types of interest by eliminating expression in non-targeted regions. More specifically, we were interested in a strategy that would enhance the neuronal specificity of transgene expression, thus facilitating exciting new functional assay platforms such as optogenetics [67].

It remains unclear whether the background patterns evident in Gal4-VP16 lines are predominantly an artifact (e.g., cryptic enhancers), promiscuous position effects, Gal4-VP16 based amplification of previously undetectable gene/enhancer activity [14], or a combination of these. Interestingly, the latter possibility is in keeping with stochastic resonance analyses suggesting many genes are active at low levels as part of natural circadian oscillations [68]. In addition, Fujimoto *et al.*, favored this explanation regarding unexpected but consistent expression patterns seen in multiple *Tg(optb.A:Gal4-VP16) *lines [14]. Combined with data presented here, this would suggest that NRSE sites are capable of delimiting endogenous enhancers, not simply reducing an artificial byproduct of the technique. Unfortunately, due to inherent ambiguities of identifying distinct regulatory elements acting on gene/enhancer traps [69,70], the transgenic lines generated here can provide only limited insight to this question. Nevertheless, the data show that regardless of the mechanism, NRSE sites serve to bias enhancer traps toward neuronal specific expression patterns, thus providing useful new resources for neurobiological research.

The addition of NRSE sites to UAS reporter lines was not sufficient to restrict reporter expression to neural tissues (Figures 3 and 7, Table 1). This is unfortunate, as NRSE-delimited UAS lines would have been useful to restrict the expression of existing Gal4-VP16 lines. How is it that NRSE sites can function to alter the expression of Gal4-based enhancer traps but not UAS reporter lines? The answer to this may be related to the fact that NRSE sites only served to bias expression; they were not 100% effective. This is in keeping with our increasing understanding of the complex multifactorial nature of gene regulation. Transcriptional activity cannot be adequately described in binary on/off terms; rather each element impinging on gene expression provides relative 'rheostat'-type modulations that are summed for final effect. Accordingly, our data suggest that, the majority of the time, REST/NRSE interactions are adequate to dampen non-neural expression when integrated with endogenous regulatory elements. Thus, in the context of an enhancer trap screen, REST/NRSE interactions are sufficient to reduce Gal4-VP16 expression to non-effective levels in non-neural populations. However, when non-neural expression of Gal4-VP16 is unchecked - i.e., when NRSE sites are solely associated with UAS reporters - the artificially enhanced strength of Gal4-VP16 transcriptional activators is the dominant element in the equation and the silencing activity of REST/NRSE is rendered ineffective.

Our findings indicate that new NRSE -delimited Gal4-VP16 driver lines will need to be derived to take advantage of the neural expression bias provided by this approach. Accordingly, we have begun a large-scale NRSE-delimited enhancer trap screen to create new Gal4-VP16 lines useful for dissecting neural circuit functions. To date, 62 NRSE-delimited KalTA4-expressing lines have been created. In a related screen, we are creating a series of NRSE-delimited LexA-based driver lines (manuscript in preparation). The use of two bipartite transgene expression systems (e.g., Gal4/UAS and LexA/LexA Operon) would allow two neuronal subpopulations to be independently manipulated. Optimally, complementary platforms of this nature could be used to differentially modulate pre- and post-synaptic elements of discrete subcircuits - a possibility that improved trans-synaptic transporters would facilitate. In addition, the use of an inducible LexA-based transactivation system in transgenic zebrafish [71] provides an additional level of temporal control over transgene expression; a strategy that increases the versatility of such systems even further.

The mechanism behind NRSE-delimited transgene expression is likely due to a repressive action of NRSF/REST in non-neural tissues [3,4]. Our data are consistent with this; a possible explanation for the reduction in non-neural phenotypes we see with NRSE lines (Figure 1S) is that, at some frequency, lines that would have expressed solely in non-neural cells are rendered silent by NRSE. However, context-dependent transcriptional regulatory functions have been proposed for NRSF/REST that span the gamut of embryogenesis: from embryonic stem cells [72], to neural progenitors [26], to differentiating neurons [28,73]. In neural lineages, NRSF/REST is thought to repress gene expression in progenitors until differentiation commences. In agreement with these models, REST expression levels decrease over time in neural lineages [26,58,59]. Conversely, NRSF/REST is thought to repress neural gene expression in non-neural cells, in keeping with NRSF/REST expression becoming progressively restricted to non-neural tissues as development proceeds. Thus, REST may play several roles during development, including repression of neuronal genes in the developing nervous system and later non-neuronal cells, and regulation of the terminal differentiation of neurons (reviewed by [74,75]). Several of our observations are in keeping with the view that REST action is more complex than simply repressing expression in non-neural cells; however, our data are not consistent with REST playing a major role in regulating neurogenesis [38].

REST is expressed nearly ubiquitously during early zebrafish development, including within the nervous system. Yet, early reporter expression (e.g., 1 to 2 dpf) is observed in the majority of NRSE-containing Gal4-VP16 driver lines (Additional file 4). This observation conflicts somewhat with reports suggesting that REST acts to repress neural differentiation programs in stem cells [72] and/or neuronal progenitors [26], which predicts that NRSE-delimited transgene expression would be limited to late neural differentiation stages. However, because NRSF/REST translation is tightly regulated [26,58,59], evaluations of REST protein expression levels and/or subcellular localization are necessary to better determine the degree to which REST expression patterns correlate to function. In addition, almost half of the NRSE-containing driver lines established to date (44%) show delayed non-neural repression of transgene expression (Figure 4). The timing of the delayed repression phenomenon is associated with a nearly two-fold increase in REST expression (Figure 5); this suggests that REST-mediated silencing requires a threshold of expression. This possibility is supported by data showing a two-fold increase in REST expression that coincides with downregulation of NRSE-containing neuronal genes in differentiating oligodendrocytes [76], and a corresponding increase in the number of REST-occupied target genes in these cells as they mature [77]. Additionally, our results are consistent with data from Kok *et al.*, showing that early neural patterning is largely unaltered in *rest *mutants [38].

It is important to note that lines displaying the delayed repression phenotype will be less useful for experiments concerning early neural development. However, their applicability to tests in late stage larvae (6 dpf) and beyond - for instance, to assay behavioral consequences of altering neuronal activity - remains viable.

To further test whether REST was required for NRSE-delimited expression patterns, we knocked down REST expression in transgenic NRCK zebrafish embryos and larvae using a previously characterized morpholino. The data showed clear evidence that when REST function is disrupted, NRSE-mediated neural expression biases are lost, with spatially expanded and temporally extended strong skeletal muscle expression seen in *rest *MO-injected NRSE-containing Gal4-VP16 driver lines (Figure 6). In addition, investigations of a *rest *mutant line (*rest^sbu29^*), generated by zinc finger nuclease targeting [78,79], showed similar results with four different NRCK transgenic lines (*gmc606, 607, 632 *and *641*) [38]. Interestingly, evidence of expanded neural expression in *rest^sbu29/sbu99 ^*mutants suggests REST can repress expression in the nervous system as well. This is in keeping with studies suggesting that REST may act to repress gene expression in neuronal subsets [28] and that REST expression is detected in neurons of certain brain regions [73]. More recently, the possibility of REST acting as a transcriptional activator has been attributed to the expression of a dominant-negative splice variant, REST4, that disrupts REST-mediated gene repression [80]. Future analyses could determine whether the presence of REST4 alters the expression of NRSE-containing transgenes.

Cell-type specific lineages are often defined by multifactorial 'codes' of overlapping subsets of transcription factors [81]. Thus, identifying individual promoter/enhancer elements providing cell-type exclusive expression patterns can be challenging. A potential strategy suggested by the data presented here is to create artificial associations between tissue-specific silencer elements and regulatory elements that target cell types of interest, albeit not exclusively. By eliminating expression in non-targeted tissues, the silencer element serves to produce the desired expression pattern; e.g., a cell-specific expression domain that might not otherwise be attainable. As an example of this approach, we are creating neuronally restricted Gal4-VP16 driver transgenic lines. These resources should facilitate the functional dissection of neural circuits in zebrafish - e.g., optogenetic [82] and/or toxin-mediated inhibition/activation of neuronal activity [8], and inducible cell ablations [46,83-85] - by restricting manipulations to targeted neuronal cell subpopulations, thus facilitating delineations of causal relationships.

## Conclusions

These studies validate the use of tissue-specific silencer elements to promote enhanced transgene expression specificity. NRSE sites served to bias the expression of trapped and defined DNA regulatory elements to the nervous system, providing a means of targeting neuronal cell subtypes by silencing expression in non-neural tissues. Transgene silencing effects were dependent on the expression of REST, in keeping with a well-characterized role of this NRSE-binding transcriptional repressor in maintaining neural-specific gene expression. Using the strategy, promoter/enhancer elements that would otherwise be too broadly expressed can be harnessed for functional assays. This approach also affords a solution to non-specific background expression issues that can compromise large-scale enhancer trap screens, as has been the case in the zebrafish field. NRSE-delimited transgenes can provide useful new tools for functional studies of the nervous system. Inclusion of bipartite expression systems, such as Gal4/UAS, ensures that a multitude of functional assays can be performed with NRSE-delimited transgenic resources. For instance, integrating new toolsets for manipulating neuronal activity or targeted cellular ablation systems into bipartite effectors will provide a versatile platform for the genetic dissection of neural circuit function. More broadly, similar genetic mechanisms may be used to reinforce expression specificity in other tissues. Thus, creating synthetic associations between endogenous regulatory sequences and tissue-specific silencer elements could provide a means of targeting unique cellular subsets for which cell-specific regulatory elements are lacking.

## Methods

### Ethics statement

This study was carried out in accordance with the recommendations in the Guide for the Care and Use of Laboratory Animals of the National Institutes of Health. An animal use protocol was approved by the Institutional Animal Care and Use Committee (Approval Identification Number: BR10-12-391) of Georgia Health Sciences University, which has an Animal Welfare Assurance on file in the Office of Laboratory Animal Welfare (Assurance Number: A3307-01). Using approved anesthetics, all efforts were made to minimize discomfort and suffering during experimental procedures.

### Zebrafish husbandry, transgenes and transgenic lines

Zebrafish were maintained using established temperature (28.5°C) and light cycle conditions (14 hours on, 10 hours off). Embryos and larvae were cultured in standard growth media supplemented with paramecia and Sera micron flake (Sera; Heinsberg, Germany) starting at 5 dpf. Previously described transgenic zebrafish strains used in this study included *Tg(elavl3:EGFP)knu3 *[56] and *Tg(14xUAS-E1b:nfsB-mCherry)c264 *[45].

Transgenes used to establish new transgenic lines during the course of these studies are diagrammed in Additional file 1. New transgenic lines include four different types of enhancer trap driver lines (two sets, ±NRSE) expressing an optimized Gal4-VP16 fusion, termed KalTA4 [43]; two motor neuron targeted (i.e., zCREST1 enhancer [35]) lines based on two multicistronic self-reporting Gal4/UAS plasmids; and two 5xUAS-based YFP reporter lines based on two different plasmids (±NRSE sequences). Two control transgenes that did not contain NRSE sites were used to establish enhancer trap lines, designated CK (*Et(cfos:KalTA4)*) and CK-5xN-14xY (*Et(cfos:KalTA4, 5xUAS-E1b:nfsB, 14xUAS-E1b:tagYFP)*). Two NRSE-containing transgenes were used to create new NRSE-delimited enhancer trap lines, designated NRCK (*Et(2xNRSE-cfos:KalTA4)*) and NRCK-5xMY (*Et(2xNRSE-cfos:KalTA4,loxP- 5xUAS-E1b:gap43-YFP-loxP)*). CREST1 enhancer containing transgenes were used to make lines designated C1CK-5xY2N (*Tg(CREST1- cfos:KalTA4,5xUAS-E1b:gap43-tagYFP-2A-nfsB)*) and NR C1CK-5xY2N (*Tg(2xNRSE-CREST1- cfos:KalTA4,5xUAS-E1b:gap43-tagYFP-2A-nfsB)*). In the CREST1 lines, a 'self-cleaving' viral peptide sequence, derived from porcine teschovirus-1 (P2A [86]), was used to promote equimolar expression of a bicistronic message [87] consisting of a YFP reporter and nitroreductase [51,88] (i.e., *YFP-2A-nfsB*). A non-NRSE 5xUAS-based reporter transgene was used to make a reporter line designated 5xMY-HMY (*Tg(loxP-5xUAS-E1b:gap43-YFP-loxP, he1a:gap43-YFP)gmc830*). In addition, a NRSE-containing 5xUAS-based reporter transgene was used to make a reporter line designated NR5xMY-HMY (*Tg(loxP-2xNRSE-5xUAS-E1b:gap43-YFP-loxP, he1a:gap43-YFP)gmc835*). All transgenes were assembled in the miniTol2 background to facilitate transgenesis efficiency [44]. A set of core cloning vectors were synthesized (GenScript; Piscataway, New Jersey, USA, or BioMatik; Wilmington, Delaware, USA) from which all transgenes were constructed. This was done to optimize codon usage (i.e., 'zebrafish-ize' codons), eliminate GC content where possible, incorporate elements promoting improved transgene stability, and provide unique cloning sites allowing functional subunits to be easily exchanged. UAS reporter transgenes generated by our laboratory were flanked by an AT-rich 'barrier' insulator sequence thought to separate methylated and unmethylated domains near CpG islands [50]. This was done in an effort to circumvent variegated transgene expression resultant to methylation-induced silencing of UAS-based transgenic lines in zebrafish, specifically demonstrated to effect expression of the *Tg(14xUAS:nfsb-mCherry)c264 *line [48,49]. Most coding sequences were followed by a rabbit β-globin intron sequence shown to improve viral and transgene expression efficiency [89] that was previously evaluated for the ability to promote mRNA stability and nuclear export of KalTA4 transgenes [43]. Finally, a transgene 'tracer' strategy was used to facilitate identification of UAS reporter transgene carriers in the absence of Gal4-VP16 expression. A 365 bp promoter of the zebrafish hatching enzyme 1a (*he1a*), which contained three regions highly conserved between *he1a, he1b *and *he2*, was used to drive expression of membrane-tagged YFP (i.e., *he1a:gap43-YFP*) in the hatching gland, a set of cells located within the yolk sac that are resorbed after hatching. This allows UAS reporter carriers to be identified by a 'temporary tracer' that fades by 4 dpf, thus does not impinge on late larval imaging experiments (see Additional file 5), unlike similar strategies using heart- and lens-specific promoters that are expressed into adulthood. Complete transgene sequences and cloning details are available upon request. Stable lines were established in the *roy orbison *(*roy*) pigmentation mutant background with Tol2-based transgenesis methods.

### RNA isolation and real-time qRT-PCR analysis

Ten wild-type embryos were collected at the indicated developmental stages and total RNA was isolated using TRIzol (Life Technologies; Grand Island, New York, USA) according to the manufacturer's protocol and treated with DNase (Promega; Madison, Wisconsin, USA) to remove genomic DNA contamination. The first-strand cDNA synthesis was performed using the SuperScript II First-Strand System (Life Technologies; Grand Island, New York, USA). qRT-PCR reactions were carried out as described previously [90,91]. In brief, cDNA amplification was performed in triplicate using the Bio-Rad iQ SYBR Green Supermix (Bio-Rad; Hercules, California, USA) on a Bio-Rad iCycler (Bio-Rad; Hercules, California, USA). Gene expression levels were normalized to β-actin by 2-ΔΔCT methods. The primers used in this study were as follows: β-actin: forward 5'- CGAGCAGGAGATGGGAACC - 3'; reverse 5'- CAACGGAAACGCTCATTGC - 3'; REST: forward 5'- GAGAGCGCAGAGAGCAACTC - 3'; reverse 5'- GCGCAGATGGTGCACTTGAA - 3'.

### Disruption of NRSF/REST expression

To knock down REST production, a previously characterized splice inhibiting morpholino targeted to the intron-exon boundary of zebrafish *rest *exon 3 (5'-GGCCTTTCACCTGTAAAATACAGAA-3') was used. The control morpholino was the standard provided by GeneTools (5'-CCTCTTACCTCAGTTACAATTTATA-3'; Philomath, Oregon, USA). Morpholinos were diluted to 0.1, 0.25, 0.5, 1 or 2 μM and a 1- to 2-nL volume injected into eggs as previously described [90]. Images from a 0.1-μM injection are shown in Figure 6. Zinc finger nuclease targeting of the *rest *locus was as previously described [38].

### Confocal imaging

All single time point and time lapse confocal imaging of transgenic zebrafish larvae was performed as previously described [83].

### Statistical analyses

Statistical comparisons were performed using an independent sample *t*-test to compare across treatment conditions, or a repeated measures *t*-test for time series data; i.e., when data from individuals were compared across time. Where symbols are present in figures, *P*-values were minimally ≤ 0.05.

## Abbreviations

2A: a porcine 2A viral peptide sequence; 2xNRSE: a tandem repeat of a 21 bp consensus NRSE site; 5xMY-HMY: *Tg(loxP-5xUAS-E1b:gap43-YFP-loxP: he1a:gap43-YFP) *transgene or transgenic reporter line (e.g.: allele number *gmc930*); 5xUAS: transgene sequence composed of five serial repeats of UAS binding sites; 14xNTR-Ch: *14xUAS-E1b:nfsB-mCherry)c264 *transgenic line; bp: base pairs; C1CK-5xY2N: *Tg(CREST1- cfos:KalTA4:5xUAS-E1b:gap43-tagYFP-2A-nfsB) *transgene or transgenic line (allele number *lmc002*); cfos: minimal promoter element from mouse *cFos *gene; CK: *Tg(cfos:KalTA4) *transgene or transgenic lines (allele numbers *gmc675-gmc699*); CK-5xN-14xY: *Et(2xNRSE-cfos:KalTA4: 5xUAS-E1b:nfsB: 14xUAS-E1b:tagYFP*) transgene or enhancer trap transgenic lines (allele numbers *gmc700-gmc724*); CREST1: highly conserved enhancer element from the zebrafish Islet-1 gene; dpf: days post-fertilization; E1b: basal promoter from carp beta-actin; Gal4: yeast transcription activator protein; Gal4/UAS: a bipartite transgene expression amplification system; Gal4-VP16: fusion protein linking the DNA binding domain of Gal4 and transcriptional activation domain of VP16; KalTA4: Gal4-VP16 fusion variant optimized for expression in zebrafish; lox: loxP recombination site; MO: morpholino-modified oligonucleotide; M-YFP: membrane-tagged yellow fluorescent protein; nfsB: bacterial gene encoding nitroreductase B; NR5xMY-HMY: *Tg(2xNRSE-loxP-5xUAS-E1b:gap43-YFP-loxP: he1a:gap43-YFP) *transgene or transgenic reporter line (e.g.: allele number *gmc932*); NRC1CK-5xY2N: *Tg(2xNRSE-CREST1- cfos:KalTA4:5xUAS-E1b:gap43-tagYFP-2A-nfsB) *transgene or transgenic line (e.g.: allele number *lmc003*); NRCK: *Et(2xNRSE-cfos:KalTA4) *transgene or enhancer trap transgenic lines (allele numbers *gmc600-gmc674*); NRCK-5xMY: *Et(2xNRSE-cfos:KalTA4: 5xUAS-E1b:gap43-YFP) *transgene or enhancer trap transgenic lines (allele numbers *gmc725-gmc774*); NRSE: neuron-restrictive silencer element; NRSF: neuron-restrictive silencing factor; qRT-PCR: quantitative reverse transcriptase polymerase chain reaction; RE1: restriction element 1; REST: RE1-silencing transcription factor; STMN2: stathmin-like 2 gene (aka SCG10); Tol2: a member of the hAT family of transposons; UAS: upstream activating sequence; VP16: viral protein 16, a strong transcriptional activator; YFP: yellow fluorescent protein.

## Competing interests

JSM and MTS have a financial interest in Luminomics, Inc., a small biotechnology company that uses the nitroreductase transgene-based system of targeted cellular ablation as a platform for studying cell-specific regeneration using models of degenerative and autoimmune diseases. JRM, M-AS and MTS are salaried employed of Luminomics. JSM receives consulting fees from Luminomics. XX, SLW, YT, MD, RWK and HIS confirm that they fdo not have any competing interests.

## Authors' contributions

XX, JRM, M-AS, and MTS created transgenic lines, and collected, analyzed and assembled the expression data. XX and SLW performed, analyzed and assembled the morpholino experiments. YT performed the qRT-PCR analysis. MD, RWK and HIS provided research materials. JSM conceived the study, and HIS, MTS and JSM participated in the design of the study. JSM drafted the manuscript with assistance from all other authors. All authors read and approved the final manuscript.

## Supplementary Material

Additional file 1 **Diagram of transgene constructs**. Schematics showing pertinent details of the transgenes tested and corresponding acronyms. Core elements include: **cfos **- minimal promoter [34]; **KalTA4 **- an optimized Gal4-VP16 fusion protein [43]; **GI **- rabbit beta-globin intron to promote mRNA stability [89]; **pA **- SV40 or bovine growth hormone polyadenylation sequences; **2xNRSE **- a tandem repeat of a 21 bp consensus NRSE site [33], **lox **- loxP recombination sites to allow transgene cassette swapping [92]; **UAS **- 17 bp upstream activator sequence [63] specific for the Gal4 DNA binding domain (with indicated number of repeats, e.g., 5x or 14x); **E1b **- a basal promoter from carp beta-actin [37]; **CREST1 **- a 800-bp enhancer element characterized as a cranial motor neuron-specific element [35]; **2A **- a porcine 2A viral peptide sequence [86] promoting equimolar expression of multicistronic messages [87]; **nfsB **- *Escherichia coli *gene encoding the prodrug converting bacterial enzyme nitroreductase (Ntr) which promotes chemically-induced cell ablation [52,53,88]; **HE **- a 365-bp promoter element from the zebrafish hatching enzyme 1a locus (*he1a*) that allows facile detection of UAS reporter lines in the absence of Gal4-VP16 driver elements (see Additional file 5). Fluorescent reporters included: **M-YFP **- a membrane-tagged (dual palmitoylation sequence from the *Xenopus gap43 *locus [93] 'enhanced' yellow fluorescent protein (EYFP); **M-tYFP **- a membrane-tagged (same as above) monomeric 'tag' yellow fluorescent protein (tagYFP); **mCherry **- a monomeric red fluorescent protein [94]. Click here for file

Additional file 2 **Enhancer trap comparisons ±NRSE**. Confocal images of an additional 12 NRCK (left box) and 6 CK (right box) lines are shown in support of the phenotypic data summarized in Figure 1S. Each line is designated by a transgenic allele number (e.g., gmc601) and with the phenotypic characterization (e.g., Neural, Mixed, Non-Neural) provided in the lower right of each image set. Additional high resolution imaging data is available on line at [47]. Click here for file

Additional file 3 **High-resolution imaging of branchiomotor neuron labeling**. (**A-E) **Confocal images of 6-dpf NRC1CK-5xY2N transgenic line (*Tg(2xNRSE-CREST1-cfos:KalTA4, 5xUAS-E1b:YFP-2A-nfsB)lmc003*) showing specific labeling of branchiomotor neuron ganglia. When NRSE sites were placed upstream of CREST1-cfos, expression became restricted to cranial motor neuron subpopulations; the expression pattern originally characterized as CREST1-specified [35]. (B, C) Motor ganglia expression included cranial nerve X (vagus, arrow in hindbrain region), VII (facial, down arrowhead), anterior and posterior V (trigeminal, up arrowhead); IV and III (trochlear and oculomotor, respectively, right arrowhead). (D, E) Unidentified descending spinal nerve. Click here for file

Additional file 4 **Early neuronal expression of NRSE Gal4 driver transgenes**. Confocal images of 2-dpf triple transgenic line (*Et(2xNRSE-cfos:KalTA4) gmc607*; *Tg(14xUAS:nfsB-mCherry)c264*; *Tg(elavl3:EGFP)knu3*) showing typical early neural expression (arrows indicate double labeled neuronal cells) of NRCK lines (NRSE Gal4 drivers). Click here for file

Additional file 5 **Hatching enzyme promoter-based transgene 'tracer'**. Stereoscope micrograph shows expression of *he1a:YFP *'tracer' transgene in 1-dpf embryos. This element allows transgenic UAS reporter lines (e.g., *Tg(loxP-5xUAS-E1b:gap43-YFP-loxP, he1a:gap43-YFP)gmc830*, shown here) to be visually sorted from non-transgenic siblings (asterisks) at embryonic to early larval stages in the absence of Gal4 driver expression. The 365 bp *he1a *promoter is robustly active (arrow) from 1 to 3 dpf, after which expression rapidly fades. Inclusion of this element in UAS reporter lines has greatly simplified maintenance of our stocks. Click here for file
