# Supplementary figures and images for: Silencer-delimited transgenesis: NRSE/RE1 sequences promote neural-specific transgene expression in a NRSF/REST-dependent manner
Source: BMC Biol. 2012 Nov 30;10:93. doi: 10.1186/1741-7007-10-93 (PMC3529185; doi:10.1186/1741-7007-10-93)

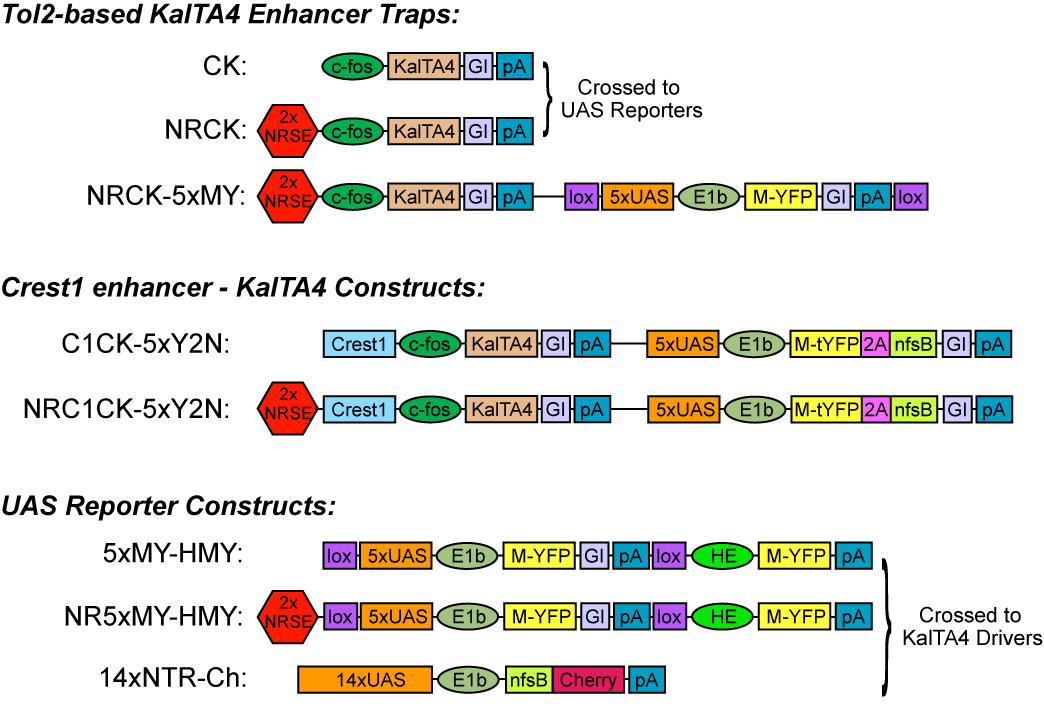

Supplement: Additional file 1 — Diagram of transgene constructs. Schematics showing pertinent details of the transgenes tested and corresponding acronyms. Core elements include: cfos - minimal promoter [34]; KalTA4 - an optimized Gal4-VP16 fusion protein [43]; GI - rabbit beta-globin intron to promote mRNA stability [89]; pA - SV40 or bovine growth hormone polyadenylation sequences; 2xNRSE - a tandem repeat of a 21 bp consensus NRSE site [33], lox - loxP recombination sites to allow transgene cassette swapping [92]; UAS - 17 bp upstream activator sequence [63] specific for the Gal4 DNA binding domain (with indicated number of repeats, e.g., 5x or 14x); E1b - a basal promoter from carp beta-actin [37]; CREST1 - a 800-bp enhancer element characterized as a cranial motor neuron-specific element [35]; 2A - a porcine 2A viral peptide sequence [86] promoting equimolar expression of multicistronic messages [87]; nfsB - Escherichia coli gene encoding the prodrug converting bacterial enzyme nitroreductase (Ntr) which promotes chemically-induced cell ablation [52,53,88]; HE - a 365-bp promoter element from the zebrafish hatching enzyme 1a locus (he1a) that allows facile detection of UAS reporter lines in the absence of Gal4-VP16 driver elements (see Additional file 5). Fluorescent reporters included: M-YFP - a membrane-tagged (dual palmitoylation sequence from the Xenopus gap43 locus [93] 'enhanced' yellow fluorescent protein (EYFP); M-tYFP - a membrane-tagged (same as above) monomeric 'tag' yellow fluorescent protein (tagYFP); mCherry - a monomeric red fluorescent protein [94]. [file 1741-7007-10-93-S1.PNG]

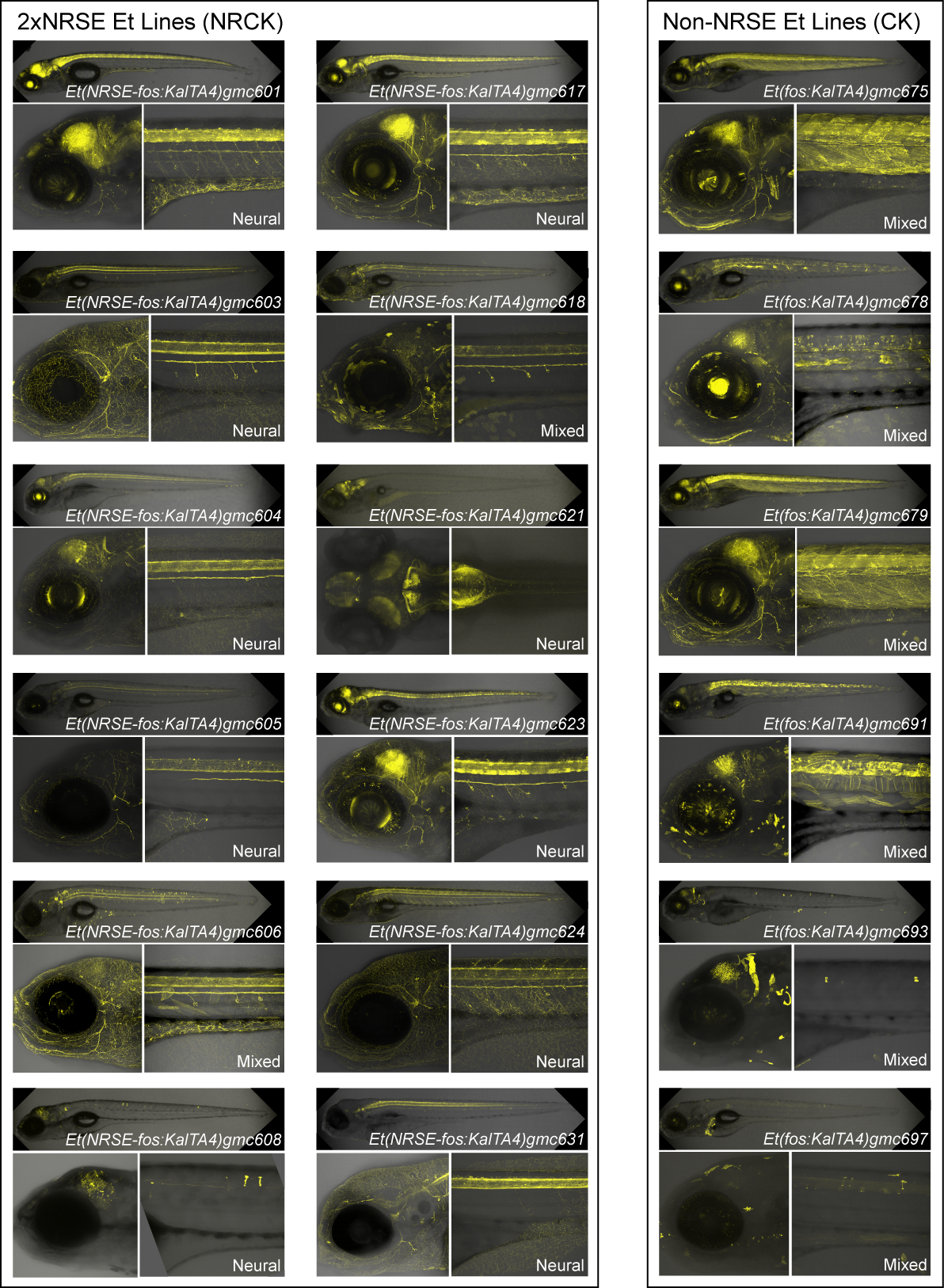

Supplement: Additional file 2 — Enhancer trap comparisons ±NRSE. Confocal images of an additional 12 NRCK (left box) and 6 CK (right box) lines are shown in support of the phenotypic data summarized in Figure 1S. Each line is designated by a transgenic allele number (e.g., gmc601) and with the phenotypic characterization (e.g., Neural, Mixed, Non-Neural) provided in the lower right of each image set. Additional high resolution imaging data is available on line at [47]. [file 1741-7007-10-93-S2.PNG]

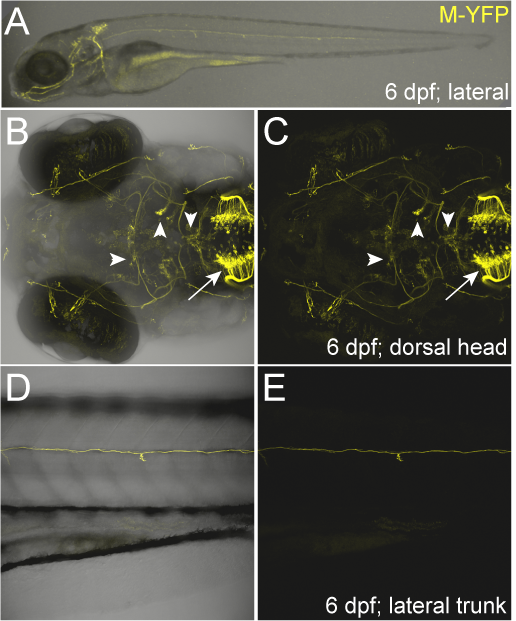

Supplement: Additional file 3 — High-resolution imaging of branchiomotor neuron labeling. (A-E) Confocal images of 6-dpf NRC1CK-5xY2N transgenic line (Tg(2xNRSE-CREST1-cfos:KalTA4, 5xUAS-E1b:YFP-2A-nfsB)lmc003) showing specific labeling of branchiomotor neuron ganglia. When NRSE sites were placed upstream of CREST1-cfos, expression became restricted to cranial motor neuron subpopulations; the expression pattern originally characterized as CREST1-specified [35]. (B, C) Motor ganglia expression included cranial nerve X (vagus, arrow in hindbrain region), VII (facial, down arrowhead), anterior and posterior V (trigeminal, up arrowhead); IV and III (trochlear and oculomotor, respectively, right arrowhead). (D, E) Unidentified descending spinal nerve. [file 1741-7007-10-93-S3.PNG]

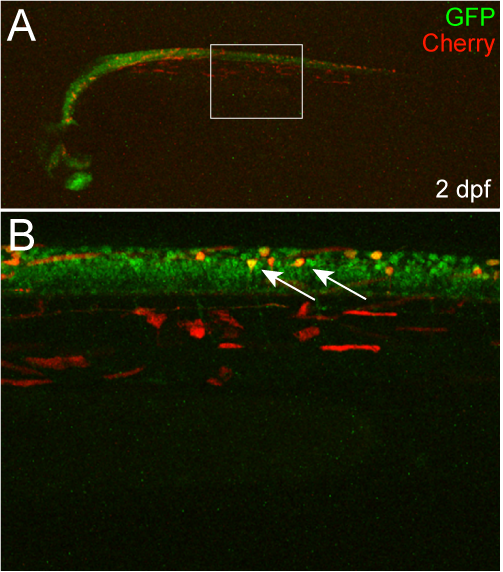

Supplement: Additional file 4 — Early neuronal expression of NRSE Gal4 driver transgenes. Confocal images of 2-dpf triple transgenic line (Et(2xNRSE-cfos:KalTA4) gmc607; Tg(14xUAS:nfsB-mCherry)c264; Tg(elavl3:EGFP)knu3) showing typical early neural expression (arrows indicate double labeled neuronal cells) of NRCK lines (NRSE Gal4 drivers). [file 1741-7007-10-93-S4.PNG]

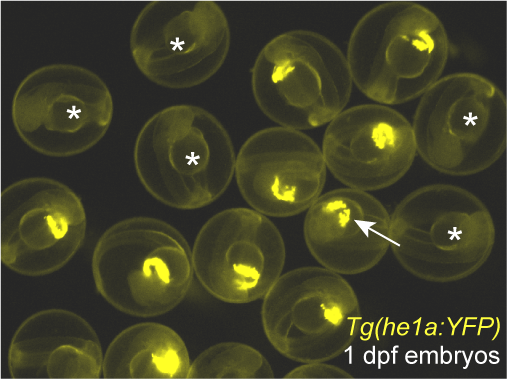

Supplement: Additional file 5 — Hatching enzyme promoter-based transgene 'tracer'. Stereoscope micrograph shows expression of he1a:YFP 'tracer' transgene in 1-dpf embryos. This element allows transgenic UAS reporter lines (e.g., Tg(loxP-5xUAS-E1b:gap43-YFP-loxP, he1a:gap43-YFP)gmc830, shown here) to be visually sorted from non-transgenic siblings (asterisks) at embryonic to early larval stages in the absence of Gal4 driver expression. The 365 bp he1a promoter is robustly active (arrow) from 1 to 3 dpf, after which expression rapidly fades. Inclusion of this element in UAS reporter lines has greatly simplified maintenance of our stocks. [file 1741-7007-10-93-S5.PNG]
